# Early Hospital Readmission (EHR) in kidney transplantation: a review article

**DOI:** 10.1590/2175-8239-JBN-2019-0089

**Published:** 2020-03-20

**Authors:** Melissa Gaspar Tavares, Helio Tedesco-Silva, Jose Osmar Medina Pestana

**Affiliations:** 1 Universidade Federal de São Paulo, Hospital do Rim, São Paulo, SP, Brasil.

**Keywords:** Patient Readmission, Kidney Transplantation, Quality Indicators, Health Care, Delivery of healthcare, Mortality, Readmissão do Paciente, Transplante de Rim, Indicadores de Qualidade em Assistência à Saúde, Prestação de Cuidados de Saúde, Mortalidade

## Abstract

Early hospital readmission (EHR), defined as all readmissions within 30 days of initial hospital discharge, is a health care quality measure. It is influenced by the demographic characteristics of the population at risk, the multidisciplinary approach for hospital discharge, the access, coverage, and comprehensiveness of the health care system, and reimbursement policies. EHR is associated with higher morbidity, mortality, and increased health care costs. Monitoring EHR enables the identification of hospital and outpatient healthcare weaknesses and the implementation of corrective interventions. Among kidney transplant recipients in the USA, EHR ranges between 18 and 47%, and is associated with one-year increased mortality and graft loss. One study in Brazil showed an incidence of 19.8% of EHR. The main causes of readmission were infections and surgical and metabolic complications. Strategies to reduce early hospital readmission are therefore essential and should consider the local factors, including socio-economic conditions, epidemiology and endemic diseases, and mobility.

## Resumo

A Readmissão Hospitalar Precoce (RH), definida como todas as readmissões dentro de 30 dias após a alta hospitalar inicial, é uma métrica da qualidade hospitalar. É influenciada pelas características demográficas da população em risco, pela abordagem multidisciplinar da alta hospitalar inicial, pelo acesso, pela cobertura e pela abrangência do Sistema de Saúde e pelas políticas de reembolso. A readmissão hospitalar precoce está associada a maior morbidade, mortalidade e aumento dos custos com saúde. O monitoramento da RH permite a identificação das fragilidades hospitalares e ambulatoriais e a implementação de intervenções corretivas. Entre os receptores de transplante renal nos EUA, a RH varia entre 18% e 47% e está associada a maior mortalidade e perda do enxerto no primeiro ano do transplante. Um estudo no Brasil mostrou uma incidência de 19,8% de RH. As principais causas de readmissão foram infecções e complicações cirúrgicas e metabólicas. As estratégias para reduzir a readmissão hospitalar precoce são, portanto, essenciais e devem considerar o ambiente local, incluindo condições socioeconômicas, epidemiologia local, doenças e mobilidade endêmicas.

## The history of early hospital readmission

The term EHR first appeared in the medical literature in 1953 examining the causes of readmission of psychiatric patients to the York Clinic, Guy's Hospital in London^1^. Thereafter, this concept was conveyed in other specialties, particularly in surgical patients, attempting to establish a causal association between the readmission and the first hospitalization, even in previously healthy patients.^2^ Further analysis suggested that only readmissions within 30 days of discharge were associated with early discharge and lack of adequate outpatient care.^3^

In 1988, the Health Care Financing Administration, a body created in 1977 to administer and supervise Medicare in the United States, required an audit of all readmissions within 30 days of discharge to determine whether the discharge was premature or whether other quality issues could be identified. This audit process was extended to hospitals serving Medicare beneficiaries and poor people, considering the lack of association between social-demographic factors and hospital readmission^4^^,^ 5. While age, sex, overall health status and type of disease, and procedures performed at admission were associated with readmission, marital status, living conditions, access to care, and insurance coverage were not^5^.

In 2009, the Center for Medicare and Medicaid Services (CMS) begin to evaluate hospital readmission rates in public institutions as part of the Annual Update Program of Data and Quality of the CMS Reporting Hospital.^6^ Beginning in 2013, hospitals that met Medicare criteria would not be reimbursed if the readmission rate was deemed excessive^6^^,^ 7. Excess readmissions rates are measured by a ratio, dividing the number of “predicted” EHR due to certain diseases (myocardial infarction, heart failure, pneumonia, chronic obstructive pulmonary disease, hip/knee prosthesis, and myocardial revascularization surgery) by the number of “expected” EHR, based on an average hospital with similar patients. A ratio greater than 1.0 indicates excess readmissions.^6^

## EHR in Brazil

In Brazil, there is no government policy linked to the Ministry of Health to verify hospital readmission in public hospitals. In the private healthcare sector, the *Agencia Nacional de Saude Suplementar* (ANS)^8^ began in December 2015 using EHR as one of the indicators of healthcare quality. Patients with obstetric complications and undergoing chemotherapy for cancer are excluded. The indicator is used for auditing and reimbursements of treatment costs. It is expected that the use of this indicator will decrease the number of readmissions in emergency services after hospital discharge. It is estimated that the rate of hospital readmission in Brazil is 19.8% with significant regional variation (North region 11.7% and South region 25.4%). Thus, EHR rate ≤20% is proposed as the goal^8^^,^ 9 ^,^ 10.

## Definition

The Ministry of Health (MS) defines hospital readmission as “*a new hospitalization in the same hospital within a certain period of time after initial hospital discharge*”^11^. EHR are defined as those occurring within 30 days because those readmissions are most likely associated with the quality of care provided during and after the previous admission^12^. Readmissions are also classified as planned and unplanned. While planned readmission usually reflect complementary diagnosis and therapy, unplanned readmissions are associated with unexpected events and, therefore, are used for research purposes^13^. Readmissions can theoretically be preventable. Quality of care during the initial hospitalization, adequate discharge planning and follow-up after discharge, and coordination between hospital and outpatient care are all associated with readmissions^14^.

## EHR as a measure of hospital care quality

EHR is a well-accepted measure of hospital care quality. A critical analysis to search for preventable causes at the time of readmission is recommended. Therefore, complications of the first hospitalization, adequacy of diagnoses and medical treatment, missing medication, proper patient education to support adherence, premature discharge, and inadequate outpatient follow-up have to be assessed^15^^,^ 16. Thus, EHR studies are essential for continuous improvement in the process of patient care 17.

The advantage of using EHR as a hospital care quality measure is the continuous monitoring of EHR rates and causes. These rates are valuable healthcare indicators used to identify and develop corrective measures for further improvements. Patients with complex and severe diseases are at higher risk for EHR, requiring a well-coordinated in- and outpatient monitoring^18^. Yet, EHR rate may not always be related to inadequate healthcare^19^. Patients with complex diseases such as congestive heart failure, asthma, and chronic obstructive pulmonary disease, have frequent exacerbations and progressions requiring hospitalization unrelated to the conducts of the healthcare provider^20^. Thus, when studying EHR, a thorough evaluation is necessary involving medical, social and welfare aspects to devise new interventions.^21^ The EHR is a parameter of care quality related to other healthcare indicators that show the population’s access to health services.

## EHR as a predictor of mortality

EHR has been associated with an increased risk of mortality. Adverse events, infections, and medication errors are the predominant risk factors^21^^,^ 22. On the other hand, in patients with chronic diseases such as congestive heart failure, liver cirrhosis, obstructive pulmonary disease, and peripheral vascular disease, EHR may indicate the natural course of progression of the condition and is associated with premature mortality^23^. In elderly patients, EHR indicates extreme vulnerability and is a strong independent risk factor for death^21^. Patients with EHR have a higher risk of developing post-hospitalization syndrome. This acquired condition is characterized by increased vulnerability due to malnutrition, changes in the sleep-wake cycle, stress, delirium, and muscular atrophy that occurs during hospitalization. At the time of hospital discharge, the physiological reserve is depleted, leading to a frail condition that increases the risk of EHR and mortality.^24^

## EHR in patients with chronic kidney diseases on dialysis

The risk of EHR is two times higher for patients with chronic kidney disease on hemodialysis compared to the general population^25^. The main causes of hospitalization are vascular access complications, hypertension, sepsis, heart failure, and acute myocardial infarction^25^. In contrast, malignancies, three or more hospitalizations in the previous year, vascular catheter access complications, intradialytic hypotension, and malnutrition are the risk factors associated with EHR. Interestingly, the reduction of prescribed drugs from admission to discharge is associated with a lower probability of EHR^26^. Patients on peritoneal dialysis are at a higher risk of EHR compared to patients on hemodialysis. The related causes are peritonitis, migration from peritoneal dialysis to hemodialysis, and incapacity to continue home peritoneal dialysis^27^. The cumulative mortality among EHR patients with chronic kidney disease is two times higher than those without EHR. EHR patients are often admitted at the emergency department, intensive care units, or in another hospital, where the previously planned care is fragmented. EHR might also be a surrogate for declining function and overall health status. Continuing patient care at hemodialysis clinics is critical to avoid further readmissions^28^^,^ 29.

## EHR of kidney transplant recipients

The first studies addressing EHR of kidney transplant recipients were published in 2008 in the United States when the CMS began evaluating hospital readmission rates at public institutions as part of the Annual Data and Quality Update Program of the CMS Report Hospital.^6^ As of 2013, hospitals that meet the Medicare criteria would not be reimbursed if the readmission rate were considered excessive. There is an intense debate as to whether this measure can be readily used among kidney transplant recipients, who in addition to having a chronic disease, which per se increases the risk of hospital readmission, underwent a surgical procedure^30^. Furthermore, most of the EHR are not potentially preventable, which confirms the severity of chronic kidney disease and the complexity of these patients.^14^^,^ 31

Using data from a cohort of 32,961 kidney transplant recipients treated by the Medicare system from 2000 to 2005 extracted from the US registry, EHR was 31%, ranging from 18 to 47% across transplant centers^32^^-^ 35. This large variation is mainly due to the characteristics of the recipient. Higher EHR rates (45.8%) were related to high risk patients, such as patients with fragility syndrome prior to transplantation. Older recipients, recipients of expanded criteria deceased donors, and prolonged delayed graft function are more prevalent in this population^32^^-^ 37. The length of stay during the transplant hospitalization varied from 5 to 8 days among US centers.^32^^-^ 35 ^,^ 38. The main causes of EHR were related to infectious complications after surgery.^33^ The independent risk factors associated with EHR were the recipient age over 40 years, black race, history of diabetes mellitus, time on dialysis, chronic obstructive pulmonary disease, recipient of expanded criteria deceased donor, no induction therapy, and length of stay longer than 5 days. In addition, in the transplanted population, as in the general population, the EHR was associated with poorer 1-year outcomes. Kidney transplant recipients who had EHR were readmitted three times more during the first year of transplantation compared to those patients who did not have EHR. More importantly, patient and graft survivals were lower in both living and deceased donor kidney transplant recipients with EHR^39^. The main causes of death among patients with EHR were cardiovascular and infections complications^40^^,^ 41.

EHR should not be seen only as an indicator of healthcare quality but also as a surrogate for mortality and graft loss during the first year post-transplant^31^. Thus, when identifying patients with a higher risk of EHR, it is possible to develop preventive intervention measures during hospitalization and outpatient follow-up.^33^

## EHR of kidney transplants recipients in Brazil

In Brazil, according to data from the Brazilian Registry of Transplants, about 5000 kidney transplants are performed every year^42^, more than 90% under the government-funded Unified Health Care System. The Brazilian Transplant System differs from that of the United States and Europe. The Brazilian allocation system considers the HLA compatibility as the primary selection criterion for kidney allocation. As a consequence, the waiting time in the list is unpredictable.^43^ Furthermore, the duration of transplant hospitalization is longer as patients are discharged after recovering from the delayed graft function period and removal of all catheters. Finally, access to hospital day or home care treatments is not universally available or affordable.

In a recent single-center Brazilian study, the incidence of EHR was 26.6% among 1175 recipients of kidney transplant between January 2011 and December 2012. The independent risk factors associated with EHR were recipient age, CMV pretransplant negative serology, induction with rabbit antithymocyte globulin, acute rejection treated during index hospitalization, and length of stay. The median time of the index hospital admission was 9 days, during which the incidence of delayed graft function was 40.2%. The high incidence of delayed graft function and the inability to discharge patients until they become dialysis-free affect EHR. Prolonged initial hospitalization provides time to diagnose and treat various complications associated with EHR. On the other hand, it increases the risk of infections, medication errors, and accidents.^44^

The main reasons for EHR were infections, surgical complications, and metabolic disturbances. Among infectious complications, CMV infection was the main cause^44^ primarily because no CMV prophylaxis was used^45^^,^ 46, which contrasts with other international centers, where the main cause of readmission is surgical. EHR was an independent risk factor for death in the first year and was associated with an lower patient and graft survivals at 12 months.^44^ CMV infection was associated with a higher risk of acute rejection, mortality, and graft loss during the first year after transplantation^47^^,^ 48 ^,^ 49.

## Interventions to reduce hospital readmission

Strategies and protocols have been developed to reduce EHR in the general population. Parker and colleagues^50^ identified interventions aiming to reduce EHR, such as discharge planning protocol, comprehensive assessments of elderly patients, and educational guidelines. The identification of patients at risk for EHR allows a targeted intervention that focuses efforts on risk factors such as medication adherence, functional status and limitations, and the need for intense follow-up.^51^ Additional interventions such as medication reconciliation, scheduled appointments after discharge, transitional care teams, and outpatient treatment follow-up are required.^52^ Establishing pharmacist support to information about medications, tracking medication compliance, and counseling about adverse drug reactions may prevent future adverse events despite the lack of a significant impact on EHR rates.^53^ None of these interventions are effective individually, providing robust support in favor of a multidisciplinary approach.^52^ Ultimately, prevention of potential readmissions improve quality of care and patient experience and is associated with reduced costs. 51

**Figure 1:**
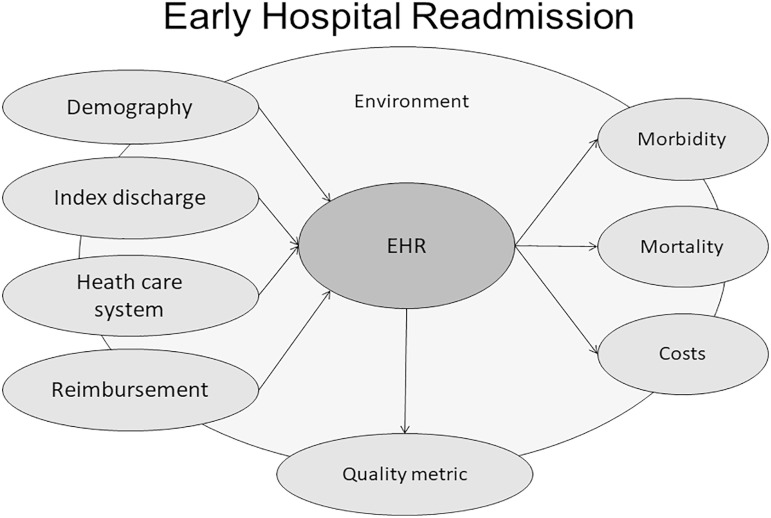
Early hospital readmission is a measure for healthcare quality. It is influenced by demographic characteristics of the population at risk, the multidisciplinary approach for hospital discharge index, the access, coverage, and comprehensiveness of the healthcare system, and reimbursement policies. Early hospital readmission is associated with higher morbidity, mortality, and healthcare costs. Strategies to reduce early hospital readmission are therefore essential and should consider the local socio-economic conditions, epidemiology, endemic diseases, and mobility.

Among kidney transplant recipients, EHR is a surrogate marker for morbidity and mortality, regardless of the demographic characteristics of the population. This marker includes not only comorbidities but also socioeconomic status, access to care, fragility level, and limited health literacy. Patients identified as vulnerable based on EHR could benefit from additional or individualized monitoring strategies, including frequent outpatient visits, phone calls, laboratory tests, monitoring of adherence, and family education^30^ (Figure 1).

## Conclusion

In the general population, EHR is a well-established measure of healthcare quality and is a robust predictor of morbidity and mortality. In the kidney transplant population, EHR is associated with mortality and graft loss as well. Measures to reduce EHR should consider multi-professional interventions considering the local demography, discharge protocols, comprehensiveness, and reimbursements of health care for the local clinical and epidemiological situation. Effective interventions will certainly reduce morbidity, mortality, and costs, increasing the quality of life of kidney transplant recipients.
